# Antagonistic interactions between maize seeds microbiome species and the late wilt disease agent, *Magnaporthiopsis maydis*

**DOI:** 10.3389/ffunb.2024.1436759

**Published:** 2024-08-07

**Authors:** Ofir Degani, Aseel Ayoub, Elhanan Dimant, Asaf Gordani

**Affiliations:** ^1^ MIGAL – Galilee Research Institute, Plant Sciences Department, Kiryat Shmona, Israel; ^2^ Faculty of Sciences, Tel-Hai College, Tel Hai, Israel

**Keywords:** seedings pathogenicity assay, *Cephalosporium maydis*, crop protection, endophytes, fungus, *Harpophora maydis*, microflora, phylogenetic analysis

## Abstract

*Magnaporthiopsis maydis* is a maize pathogen that causes severe damage to commercial corn fields in the late growth stages. Late wilt disease (LWD) has spread since its discovery in the 1960s in Egypt and is now reported in about 10 countries. The pathogen has a hidden endophytic lifecycle in resistant corn plants and secondary hosts such as green foxtail, watermelon lupin and cotton. At the same time, it could be an opportunist and hinder the host development under the right conditions. This study uncovered *M. maydis* interactions with newly identified maize endophytes. To this end, six fungi were isolated from the seeds of three sweet corn cultivars having varying susceptibility to LWD. These isolates were identified using colony morphology and microscopic characterization, universal internal transcribed spacer (ITS) molecular targeting and phylogenetic analysis. Most of them belonged to pathogenic species. Compared to three previously identified bioprotective microorganisms, the new species were tested for their ability to secrete metabolites that repress *M. maydis in vitro* and to antagonize it in a solid media confront test and a seedlings pathogenicity assay. The opportunistic fungal species *Aspergillus flavus* (ME1), *Aspergillus terreus* (PE3) and the reference biocontrol bacteria *Bacillus subtilis* (R2) achieved the highest *M. maydis* inhibition degree in the plates tests (74-100% inhibition). The seedlings’ pathogenicity assay that predicts the seeds’ microflora resistance to *M. maydis* highlighted the bio-shielding potential of most species (23% or more epicotyl elongation over the infected control)*. Fusarium* sp. (ME2) was the leading species in this measure (43% enhancement), and *B. subtilis* gave the best protection in terms of seeds’ germination (50%) and sprouts’ biomass (34%). The results of this study could enhance our understanding of the pathobiome’s role in the context of LWD and represent a first step in using the seeds’ natural protective microflora to develop novel management strategies.

## 1 Introduction

*Maize* (corn, *Zea mays L*.) is considered one of the world’s most influential human food sources and the third leading crop after wheat and rice (Zhao et al., 2017). Additionally, many countries have been growing corn as an essential animal feed source and for other uses. Maize late wilt disease (LWD) has spread since its discovery in the 1960s in Egypt (Sabet et al., 1961; Samra et al., 1962). It has been reported in 10 countries (Degani, 2021), with a higher incidence in Israel (Degani and Gordani, 2022), Egypt (El-Shabrawy and Shehata, 2018), India (Rakesh et al., 2022), Spain (Ortiz-Bustos et al., 2019) and Portugal (Patanita et al., 2020). However, the disease’s prevalence and impact are expected to increase due to global warming (Pecsi and Nemeth, 1998; Degani, 2021).

The pathogen *Magnaporthiopsis maydis* (former names *Cephalosporium maydis* and *Harpophora maydis*) *(* Samra et al., 1963; Saleh and Leslie, 2004; Luo and Zhang, 2013) causes severe damage to corn fields in the late growth stages. *M. maydis* is a potentially high-risk, hemibiotroph, seed-borne (Michail et al., 1999) and soil-borne phytopathogen (Tej et al., 2018). It is reported as being an almost asymptomatically endophyte in LWD-resistant corn cultivars (Kumar et al., 2021). It can also survive in secondary host plants such as lupine (*Lupinus termis* L.) (Sahab et al., 1985), cotton (*Gossypium hirsutum*) (Sabet et al., 1966), green foxtail (*Setaria viridis*) and watermelon (*Citrullus lanatus*) (Dor and Degani, 2019). Yet, it may become pathogenic and cause disease in some alternative hosts under certain conditions (encouraging host and environmental states) (Degani et al., 2022a). Without the host plant, the pathogen can spread and survive through infected soils and crop residues (Sabet et al., 1970).

In susceptible sweet corn hybrids, LWD is characterized by a relatively rapid wilting of the above soil parts that usually occurs 60-80 days from seeding, from before the flowering stage (tasseling) to physiological maturation (Darwesh and Elshahawy, 2023). Dehydration signs start to appear nearly 50 days post-sowing, progressing from the lower stem and leaves to the plant’s upper part, potentially damaging the cobs (Johal et al., 2004). In severe cases, the disease can cause nearly total dehydration and yield loss (El-Naggarr et al., 2015; Degani et al., 2020). Nevertheless, most outbursts of LWD have resulted in 30-50% economic losses (El-Shehawy et al., 2014; Sunitha et al., 2020).

Presently, the primary approach for LWD management is cultivating disease-resistant corn varieties (Kumar et al., 2021). This strategy is economical, eco-friendly and compatible with any growth method but requires constant effort to scan and identify new resistant corn hybrids. Moreover, the continual growth of a specific resistant maize cultivar for several seasons could ultimately lead to a selection pressure that would, in the long run, lead to an outburst of aggressive virulent *M. maydis* strains capable of causing the disease, as previously reported in Egypt, Spain and Israel (Zeller et al., 2002; García-Carneros et al., 2011; Ortiz-Bustos et al., 2015; Shofman et al., 2022).

Over the years, many strategies and control methods have been tested for LWD. A traditional approach involving chemical pesticides has produced promising results (Singh and Siradhana, 1989; Ghazy et al., 2017; Degani et al., 2018; Degani et al., 2020). Yet, public concern regarding this method’s health and environmental risks encourages searching for more eco-friendly solutions. Moreover, using a single ingredient-based chemical pesticide to protect the crops, as suggested lately (Degani and Gordani, 2022), may lead to fungal resistance development and effectiveness losses (Corkley et al., 2022). Specifically, this concern is regarding Azoxystrobin, an ingredient known to be one of the most effective fungicides to control LWD (Degani et al., 2014; Degani et al., 2019). The reason is that there is a high risk of emerging Azoxystrobin-resistant *M. maydis* strains as it occurs in other pathogens (Avila-Adame and Koller, 2003; Fernández-Ortuño et al., 2010; Castroagudín et al., 2015).

Therefore, significant research on alternative LWD management has suggested several impactable options, including biological methods (Ghazy and El-Nahrawy, 2020; Degani and Dor, 2021; El-Naggar and Yassin, 2024), agrotechnical methods such as balanced soil fertility (Samra et al., 1966; Singh and Siradhana, 1990) and watering (Singh and Siradhana, 1987a), solar heating (Fayzalla et al., 1994) and allelochemical methods (Tej et al., 2018). One focused research effort addresses the identification of potential antagonistic endophytes in commercial corn hybrids, using them as biocontrol agents to shield plants from the pathogen (Degani et al., 2021).

Obtaining new and effective environmentally-friendly LWD control options is a continuous scientific aim. Specifically, the full potential protective ability of endophytes in the maize plant’s microbiome to resist LWD is yet to be revealed. In addition, the intriguing interactions of *M. maydis* and other pathogens in the maize’s pathobiome could play a crucial role in the disease outcome and the effectiveness of the control treatments. Plants are threatened by diverse pathogen species living in complex communities that can enhance diseases or oppress one another.

The classic triangle in phytopathology requires the interaction of a susceptible host, a virulent pathogen and an environment favorable for disease development (Francl, 2001). The plant microbiome affects both host resistance and the plants’ close surroundings. These microorganism communities inhabit the plant’s rhizosphere (the roots’ nearby habitat) or phyllosphere (the plant’s aboveground habitat), and comprise opportunistic pathogens and non-pathogenic members that may protect the plant from pathogens (Compant et al., 2019). They are formed by bacteria and fungi inhabiting the same ecological niche and cooperating or competing for the same plant resources. Plant-friendly endophytes can now provide new ways of controlling plant diseases.

Studying the role of maize endophytes in restricting the impact of *M. maydis* is an essential initial step towards developing a new, environmentally-friendly control interface based on strengthening the plant’s microbiome. The protective partners in the natural microflora of maize seeds can reduce the pathogen’s development and spread within the host roots, improve the plant’s immunity to diseases and enhance growth indices. Seed treatment as a management protocol is considered economically feasible and easily applied, and could be integrated with other practices, regardless of the cultivation and irrigation methods.

In the current study, we hypothesized that fungal and bacterial microflora, natural inhabitants of various maize grains, may positively impact plant health and serve as a fundamental front barrier against the late wilt pathogen. Therefore, non-pathogenic species can be used as bio-pesticides for sustainable agriculture. In contrast, virulence species in the maize seeds’ microbiome can affect LWD in intricate ways. To test this hypothesis, we isolated microorganisms from several maize cultivars’ grains, identified them using colony morphology, microscopic traits and molecular characteristics, and tested them against *M. maydis* on media plates. Subsequently, microorganisms capable of restricting pathogen growth by direct contact or by secreting antifungal metabolites into the medium were applied to protect seedlings at the sensitive pathogen-invasive phase.

## 2 Materials and methods

### 2.1 Preparation of the maize grains

To isolate new endophytes from sweet corn seeds, we choose cultivars varying in susceptibility to LWD. All seeds were commercial and were provided courtesy of the seed companies listed in **Table 1**. Seeds were pretreated by the companies with Captan (cis-N-trichloromethylthio-4-cyclohexene-1,2-diacarboximide) according to Israel’s Plant Protection and Inspection Services (PPIS) regulations. Before being subjected to endophyte isolation, the grains were rinsed with tap water and soap while stirring 4-5 times and changing the water until the seed coating was washed. The seeds were then disinfected externally using 1% sodium hypochlorite (NaOCl) for 1 min and sterilized twice with 70% ethanol. Subsequently, the seeds were rinsed twice in sterile double-distilled water (DDW). Finally, they were dried in a biological hood on sterile paper for two hours.

**Table 1:** List of maize cultivars tested for the presence of endophytes.

Cultivar	Type	Seed Company	Supply Company	Degree of LWD Sensitivity ^1^
**Megaton**	Sweet	Zeraim Gedera-Syngenta, Kibbutz Revadim, Israel	Hazera Seeds Ltd., Berurim MP Shikmim, Israel	Hypersensitive
**Prelude**	Sweet	SRS Snowy River Seeds, Australia	Green 2000 Ltd., Israel	Sensitive
**Royalty**	Sweet	Pop Vriend Seeds B.V., Andijk, The Netherlands	Eden Seeds, Reut, Israel	Resistant

^1^According to (Degani et al., 2020; Degani et al., 2022b).

### 2.2 Isolation and identification of endophytes from the maize grains

The endophytes Isolation and identification method was previously described in (Degani et al., 2021). The corn grains were cut lengthwise using a sterile scalpel and placed on potato dextrose agar (PDA; Difco Laboratories, Detroit, MI, USA) growth plates, with the cutting surface downwards. The Petri plates (90-mm-diameter plate with 10 half-seeds each) were incubated at 28 ± 1°C in the dark for 2-3 days. The developed endophytes (fungi and bacteria) were isolated into new PDA plates and grown for 5-6 days under the above conditions. The isolates (ca. 30) were divided into groups having similar colony characteristics. Transfer to new plates was performed for representative isolates from each group, and the process continued until pure colonies were obtained. Each colony was transferred to a new plate by taking a small agar disk from the colony’s edge and closely observing its appearance as it grew.

The mycelial mats or conidia were gently removed from the plates, and a small portion was mixed with 10 µL of either potato dextrose broth (PDB) or DDW. These mixtures were then applied onto sterile glass slides for microscopic examination using a light microscope at a magnification of 400× without staining. The microscopic identification of the species (mainly spores and conidiophores characteristics) was conducted using taxonomic keys.

### 2.3 Molecular identification of the endophytes

DNA was extracted from the mycelia of PDA-grown endophyte colonies using the Master Pure Yeast DNA Purification Set Kit (Sigma, Rehovot, Israel). Molecular identification by PCR and sequencing was performed by targeting the endophyte’s small subunit ribosomal RNA gene (16s small rRNA gene) using the universal internal transcribed spacer (ITS4 and ITS5) primers (**Table 2**). PCR was done using the Rapidcycler (Idaho Technology, Salt Lake City, UT, USA) in a total volume of per reaction; of each primer (concentration of ), of commercial reaction mixture RedTaq^®^ ReadyMix (Sigma, Rehovot, Israel), of template DNA and autoclaved DDW. PCR conditions were 94°C for 2 min, 30 rounds of 94°C for 30 sec, 55°C for 30 sec, 72°C for one min, and a finishing step of 72°C for 5 min (Degani et al., 2021). The PCR products were kept at 4°C until use. PCR products were sequenced in the ITS4 forward and ITS5 reverse directions by Hy Labs (Rehovot, Israel). Sequences were used to conduct a homology search against GenBank using the BLASTN tool (National Center for Biotechnology Information, Bethesda, MD, USA, at: http://www.ncbi.nlm.nih.gov, accessed on 15 May 2024). For the *Fusarium* species identification, the Fusarioid-ID database pairwise alignment (at www.fusarium.org, accessed on 15 May 2024) was used.

**Table 2:** The primers used in this study for the endophytes’ identification.

Primer	Sequence	Uses	Amplification
ITS4 ITS5	5′-TCCTCCGCTTATTGATATGC-3′ 5′-GGAAGTAAAAGTCGTAACAAGG-3′	PCR target gene	Small subunit ribosomal RNA gene 560 bp

Sequence comparison of the ITS gene and phylogenetic tree construction were performed using the SeaView version 5.0 software (http://doua.prabi.fr/software/seaview, accessed on 15 May 2024) (Gouy et al., 2010). Sequences were aligned using the CLUSTALW program (https://www.genome.jp/tools-bin/clustalw, accessed on 15 May 2024), and the similarity percentages between sequences in the phylogenetic tree were constructed using the parsimony DNA-level algorithm (dnapars) method with the default parameters, ignore positions with gaps and bootstrap with 1000 replications. The parsimony score for each tree is the sum of the smallest number of substitutions needed for each site. The method assumes that the tree with the lowest parsimony score, the simplest of the set, is most likely to be correct. The ITS genes from reference strains found in a previous study (Degani et al., 2021) and others selected from the GenBank were incorporated into the analysis to assist the taxonomic assignment.

### 2.4 Endophytes’ metabolites assay

In a non-indirect method, the growth restriction of *M. maydis* was tested using endophyte-secreted metabolites (Degani and Dor, 2021). Ten 6-mm-diameter agar disks were cut from the culture margins of each endophyte isolate colony (grown for 4-6 days at 28 ± 1°C in the dark) and added to 150 mL sterile potato dextrose broth (PDB) in a 250 mL Erlenmeyer flask. Cultures were incubated at 28 ± 1°C in a rotary shaker at 150 rpm for six days in the dark. The liquid medium of each endophyte culture was filtered through two Whatman No.1 filter papers using a Büchner funnel. To prepare endophytes’ extrolites-based solid growth medium, 5.85 grams of PDA powder was added to a 300 mL bottle, mixed with each filtrated liquid growth medium, and the pH was adjusted to 5-5.3 with NaOH (PDB pH value). The liquid medium was autoclave sterilized for 30 min. Then 25 mL was poured into a 90 mm-diameter Petri dish (five repeats for each endophyte isolate) and dried in a biological hood for 24 h. *M. maydis* 6-mm-diameter agar disks were cut from a culture’s margins, and one colony agar disk was placed on each dish. PDA plates without extrolites were used as a control.

### 2.5 Plates’ confrontation assay

Selected endophytes were identified by their biocontrol potential to restrict *M. maydis* growth in a direct confront test according to (Degani et al., 2021). Such potential includes their antifungal compounds’ secretion, direct hyphae contact growth inhibition, or the ability of the endophyte to grow on top of the *M. maydis* colony surface. The mycoparasitism test was performed by placing endophyte colony agar disks (6 mm in diameter cut from culture margins) on a 90-mm-diameter PDA culture Petri dish in front of similar disks from *M. maydis*. Plates were labeled and incubated at 28 ± 1°C in the dark. The interactions between *M. maydis* and each isolated endophyte were documented and photographed after seven days. Endophytes that managed to restrict *M. maydis* growth were marked as having a microparasitic potential. Each endophyte was tested in five repeats.

### 2.6 Percentage of inhibition of *M. maydis*

After six days of growth, the inhibition for the metabolites assay experiment (Section 2.4) was calculated using (%I) = [((A+B)/2)x100/R], where R = radial growth of the control; A+B = radial growth of treatment. The percentage of *M. maydis* inhibition for the confrontation assay experiment (Section 2.5) was calculated using (%I) = [(R-DM)x100/R], where R = radial growth of the control; DM = radial growth of the treatment (Chagas et al., 2022). The isolated endophyte with a high %I was considered an efficient antagonist toward the pathogen *M. maydis*.

### 2.7 Seedings pathogenicity assay

Endophytes-treated maize seeds were tested in a pathogenicity assay to evaluate the level of *M. maydis* bio-shielding (as in (Degani et al., 2021)). The sweet corn Prelude cv. seeds were rinsed in DDW, soaked in 1% NaOCl for 1 min, and then rinsed twice with DDW. The endophytes’ cultures were grown in a liquid PDB medium, as described in Section 2.4. The cultures were ground in a blender for 2 minutes to obtain a blend of short mycelial segments. This mycelia and growth medium combination was used to prepare the seed-coating suspension. Sixty seeds were then soaked for 15 min in the growth medium of each endophyte culture (150 mL) mixed with 7 ml of Tween 80. Ten treated grains were transferred to a 90 mm-diameter Petri dish in which sterile Whatman paper was soaked in 5 ml DDW water. A 6-mm-diameter agar disk of *M. maydis* (grown previously on PDA in the dark at 28 ± 1°C) was added to each plate center. A sterile 6-mm-diameter PDA disk was added to the control group. Three ml DDW was added to each plate every three days to maintain moisture and to allow efficient seed germination and pathogen inoculation. After six days of incubation at 28 ± 1°C in the dark, the seeds were photographed, and their germination percentages, epicotyl length and fresh biomass were assessed.

### 2.8 Statistical analysis

The data from the *in vitro* plates and seedlings pathogenicity assays were analyzed using Microsoft Excel (version 2401 Build 16.0.17231.20290) and GraphPad Prism software (version 9.5.1.733, GraphPad Software Inc., San Diego, CA, USA). Statistical analysis included a Shapiro-Wilk normality test followed by a one-way analysis of variance (ANOVA) and a post-doc of Dunnett’s test (which compares experimental groups to a single control group) or the Fisher’s Least Significant Difference (LSD) test, and at a significance level of *p* < 0.05.

## 3 Results

**Figure 1:**
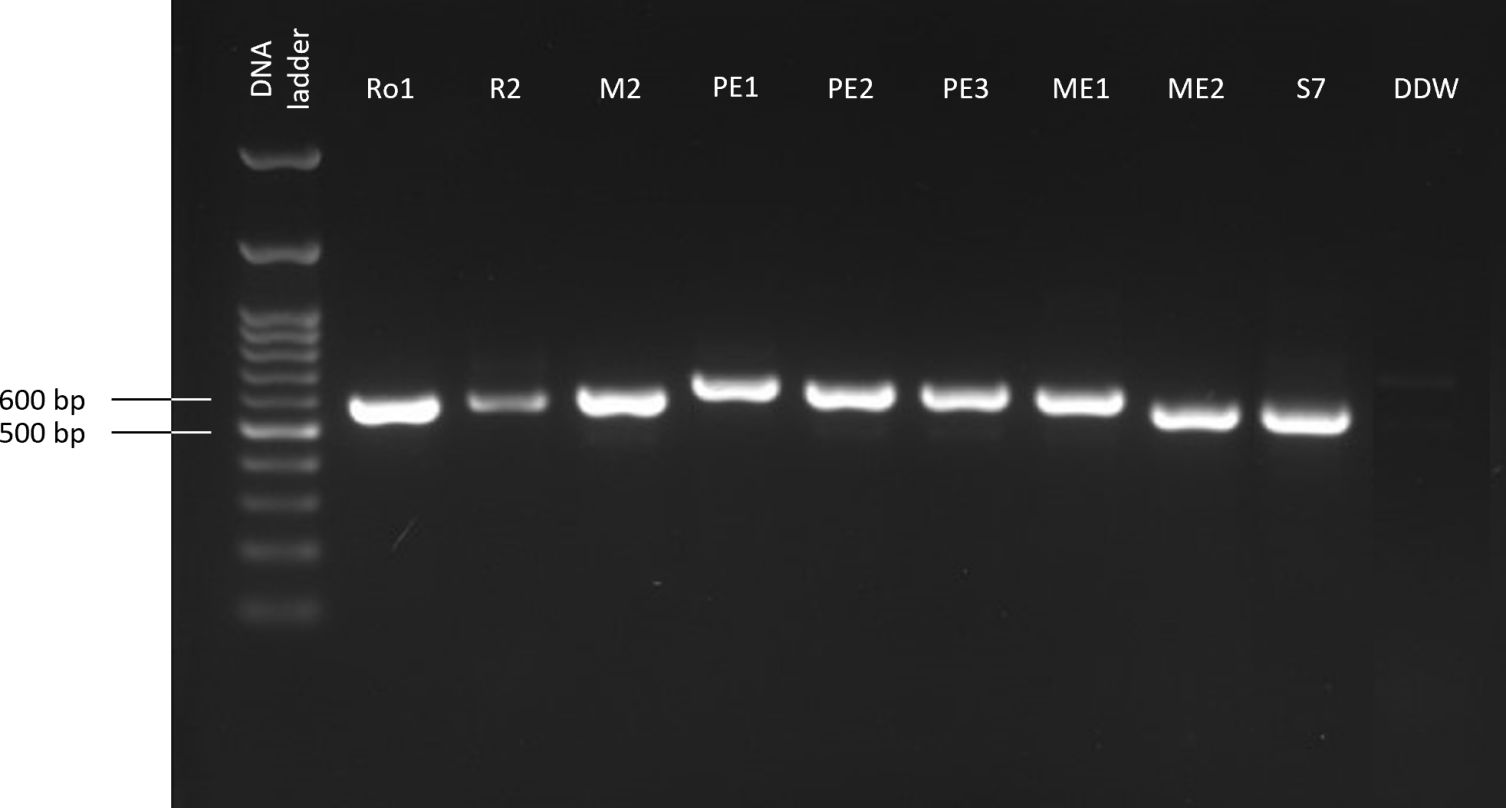
PCR-based molecular identification of the maize seeds’ endophytes. The gel presented is cropped and rearranged to improve the presentation’s clarity and conciseness. The full-length gel is presented in **Supplementary Figure S1**. The amplified product (ITS, targeting the small subunit ribosomal RNA gene using the universal internal transcribed spacer) was detected by gel electrophoresis. The negative control (DDW) includes sterile twice-distilled water instead of the DNA template.

**Figure 2:**
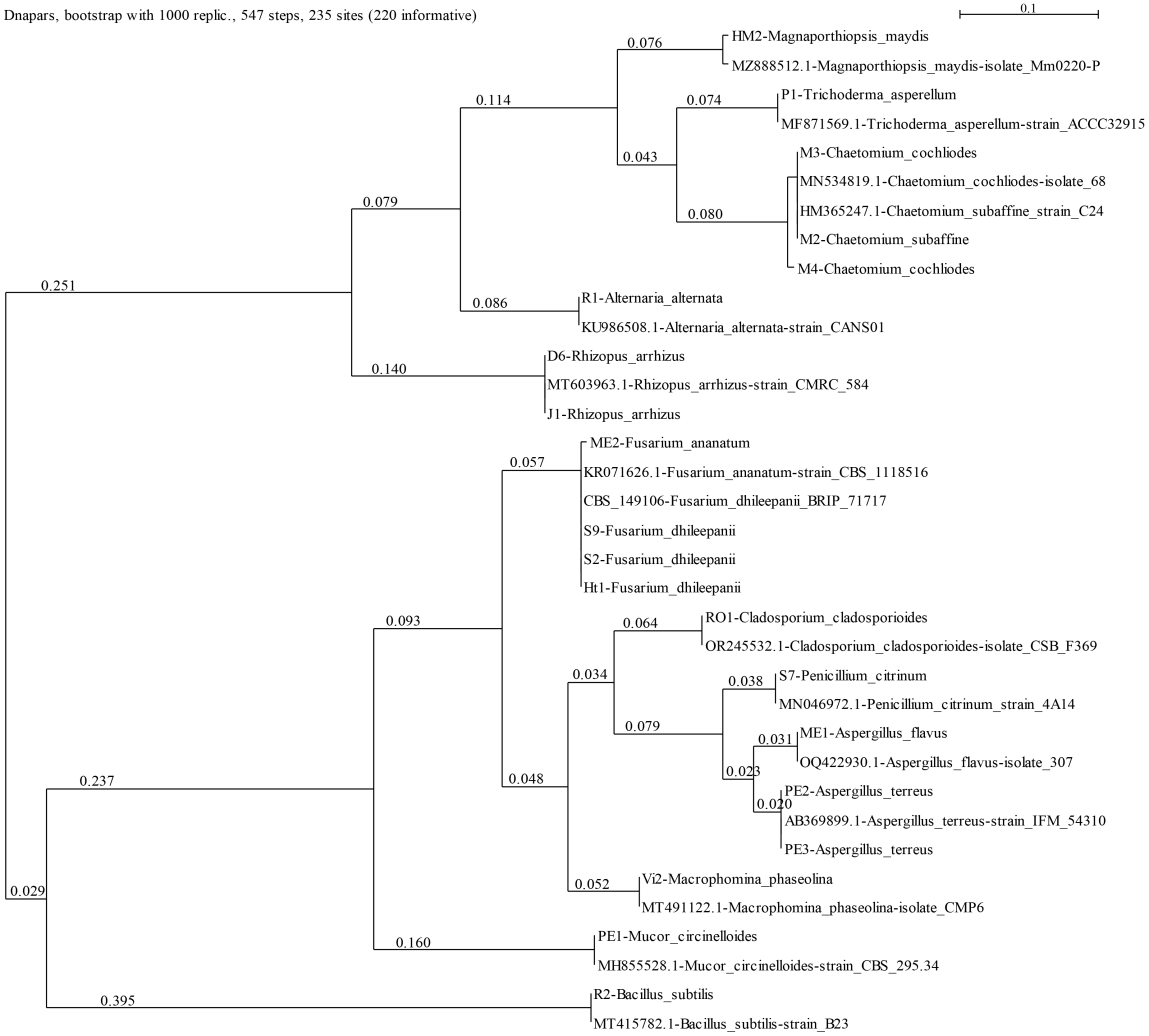
Phylogenetic analysis of the ITS4 gene of the endophytes’ isolates (presented in **Table 3**). The SeaView version 5.05 program (http://doua.prabi.fr/software/seaview, accessed on 15 May 2024) generated the phylogenetic tree using the parsimony DNA-level algorithm (dnapars)-based method with the default parameters and bootstrap with 1000 replications. The analysis contains the ITS gene from reference strains from a previous work (Degani et al., 2021) to present all maize seeds’ endophyte species identified in Israel so far and others selected from the GenBank to assist the taxonomic assignment. These can be identified by their NCBI accession number. The scale describes the genetic resemblance percentages between sequences. The statistical measures (bootstrap support and posterior probability) for each node are displayed.

The current work aimed at isolating and identifying maize seeds’ endophytes and exploring their bioprotective potential against the late wilt (LWD) pathogen *M. maydis*. First, six fungal species were isolated and identified molecularly (**Figure 1**; **Table 3**). These selected endophytes belong to the genus *Aspergillus*, *Fusarium*, *Mucor* and *Cladosporium*. The homology search using the BLASTN and Fusarioid-ID tools resulted in 95.30-100% similarity to GenBank species. The isolates’ identity (confirmed by colony morphology and microscopic traits) was verified by phylogenetic analysis (**Figure 2**; **Supplementary Figure S2**). The phylogenetic tree consists of all maize seeds’ endophyte species identified in Israel so far and provides a more comprehensive understanding of these endophytes’ communities. It may be drawn from the analysis that the species composition is usually not linked to a specific maize cultivar. One exception is the *Chetomium* species, which was isolated from Megaton seeds (a highly LWD susceptible genotype). The endophytes’ population is divided into two major branches. The first includes two sub-branches: the *Rhizopus* and *Alternaria* sub-branch; and the *Magnaporthiopsis*, *Trichoderma* and *Chetomium* sub-branch. The second consists of *Mucor circinelloides* and *Macrophomina phaseolina*, which are separate from the *Penicillium* and *Aspergillus* species group and the *Cladosporium* and *Fusarium* spp. group.

**Table 3:** Endophytes included in this study and their metabolites and confront assays results^a^.

Desig.	Maize Cultivar	Primer	Species	Class.	NCBI/Fusarioid-ID Accession	Percent identity	Metabolites’ inhibition^b^	Confront assay winner^c^
**M2 ^d^**	Megaton cv.	ITS 4	*Chaetomium subaffine*	Fungi	HM365247.1	100%	8.24%	(+)
		ITS 5	*Chaetomium cochliodes*	Fungi	MN534819.1	98.33%		
**S7 ^d^**	Simon cv.	ITS 4	*Penicillium citrinum*	Fungi	MN046972.1	99.57%	1.23%	(++)
		ITS 5	*Penicillium citrinum*	Fungi	OP237262.1	98.85%		
**R2 ^d^**	Royalty cv.	ITS 4	*Bacillus subtilis*	Bacteria	MT415782.1	99.06%	82.89%	(++)
		ITS 5	*Bacillus subtilis*	Bacteria	MT415782.1	99.06%		
**ME1**	Megaton cv.	ITS 4	*Aspergillus flavus*	Fungi	OQ422930.1	99.48%	73.64%	(++)
		ITS 5	*Aspergillus flavus*	Fungi	KY006838.1	95.30%		
**ME2**	Megaton cv.	ITS 4	*Fusarium ananatum*	Fungi	CBS 118516	97.74%	43.64%	(+)
		ITS 5	*Fusarium beomiforme*	Fungi	CBS 100160	99.16%		
**PE1**	Prelude cv.	ITS 4	*Mucor circinelloides*	Fungi	MH855528.1	99.10%	0.0%	(++)
		ITS 5	*Mucor circinelloides*	Fungi	MT603942.1	99.66%		
**PE2**	Prelude cv.	ITS 4	*Aspergillus terreus.*	Fungi	AB369899.1	97.06%	69.35%	(++)
		ITS 5	*Aspergillus terreus.*	Fungi	MH472622.1	98.50%		
**PE3**	Prelude cv.	ITS 4	*Aspergillus terreus.*	Fungi	MK108382.1	98.63%	100%	(++)
		ITS 5	*Aspergillus terreus.*	Fungi	MH472622.1	96.42%		
**RO1**	Royalty cv.	ITS 4	*Cladosporium cladosporioides*	Fungi	KU680349.1	100%	0.0%	(-)
		ITS 5	*Cladosporium cladosporioides*	Fungi	MG755208.1	96.43%		

^a^Maize seeds’ endophytes molecular identification and their metabolites and confront assay results summary. Identifying the isolates relies on the highest sequence similarity scores of the ITS regions using the NCBI GenBank BLASTN search (National Center for Biotechnology Information, Bethesda, MD, USA, at: http://www.ncbi.nlm.nih.gov, accessed on 15 May 2024). For the Fusarium species identification, the Fusarioid-ID database pairwise alignment (at www.fusarium.org, accessed on 15 May 2024) was used.

^b^The first assay tested the endophyte’s secreted metabolites’ ability to restrict M. maydis growth on a solid potato dextrose agar (PDA) medium. Antagonist endophytes with high inhibition (%I) scores (above 60%) were marked as winners in the metabolites’ assay.

^c^Second, in the directly confront assessment on PDA, the endophytes were classified as strong (++), moderately strong (+) or weak (-) antagonists according to their ability to suppress M. maydis growth.

^d^The top three species were isolated and identified in a previous study (Degani et al., 2021) and used here for comparison.

The newly identified endophyte species’ bioprotective potential was tested compared to three previously recognized species (Degani et al., 2021) by assessing their secreted metabolites’ inhibition capability and direct confront antagonism against *M. maydis* (**Table 3**). These tests are presented in detail below.

**Figure 3:**
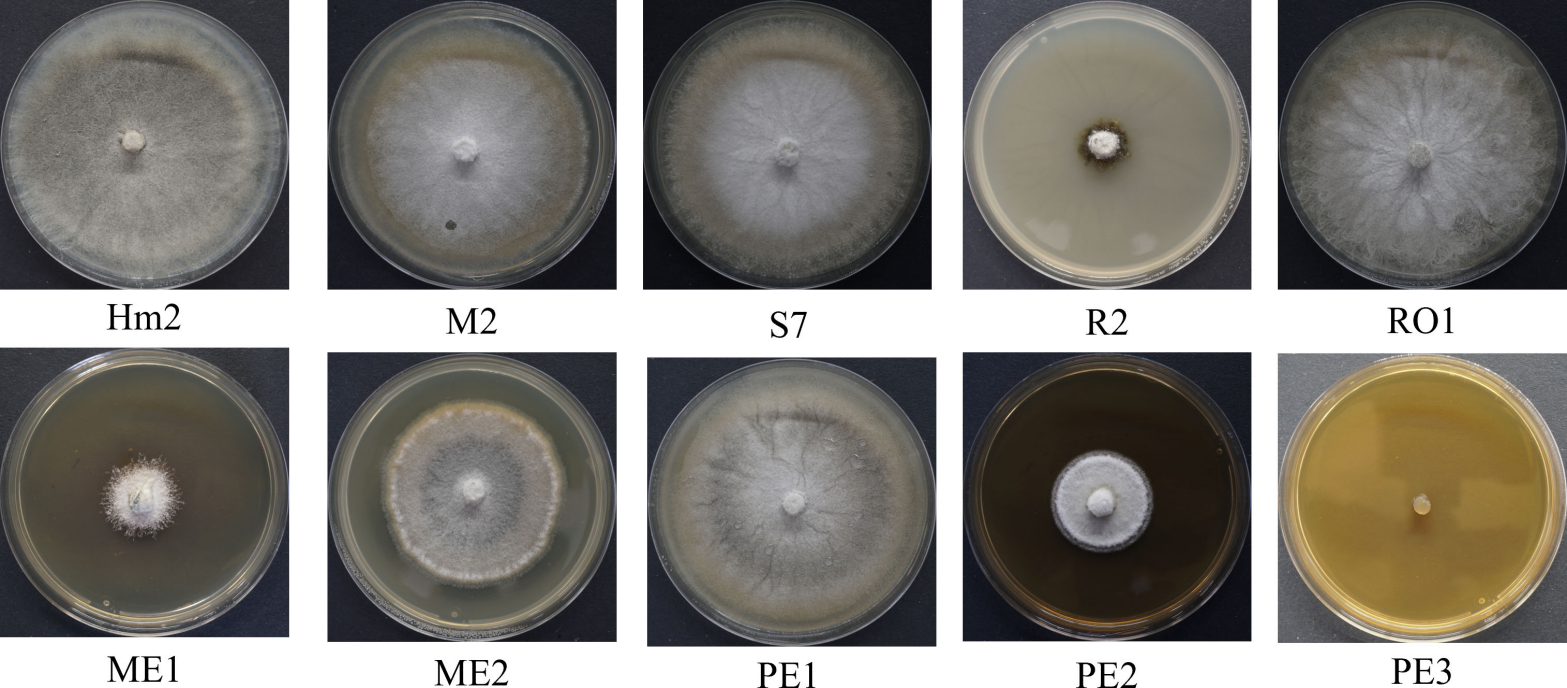
Endophyte’s secreted metabolites inhibition assay to identify *M. maydis* antagonism. This non-directed method identifies the interaction between the pathogen *M. maydis* and selected endophytes listed in **Table 3**. The control was an *M. maydis* (Hm2 isolate) culture grown on a regular PDA medium (without extrolites). Growth inhibition of *M. maydis* after six days of incubation indicates the endophyte secretion antifungal properties. Each photo represents five repeats for each seed colonist’s assay.

The non-directed method assesses the antagonism derived from the secretion of antipathogenic compounds that block *M. maydis* colony growth. Summarizing the qualitative results (**Figure 3**) revealed that four of the isolates were able to repress the pathogen through the secretion of such metabolites into the growth medium: the bacteria *Bacillus subtilis* (R2) and the three fungal colonists – *Aspergillus terreus* (PE2, PE3), and *Aspergillus flavus* (ME1). Specifically, one species, *A. terreus* (PE3), excelled in this test and completely inhibited the pathogen. In contrast, in the presence of *Cladosporium* sp. (RO1) extrolites, *M. maydis* grows without apparent interference.

**Figure 4:**
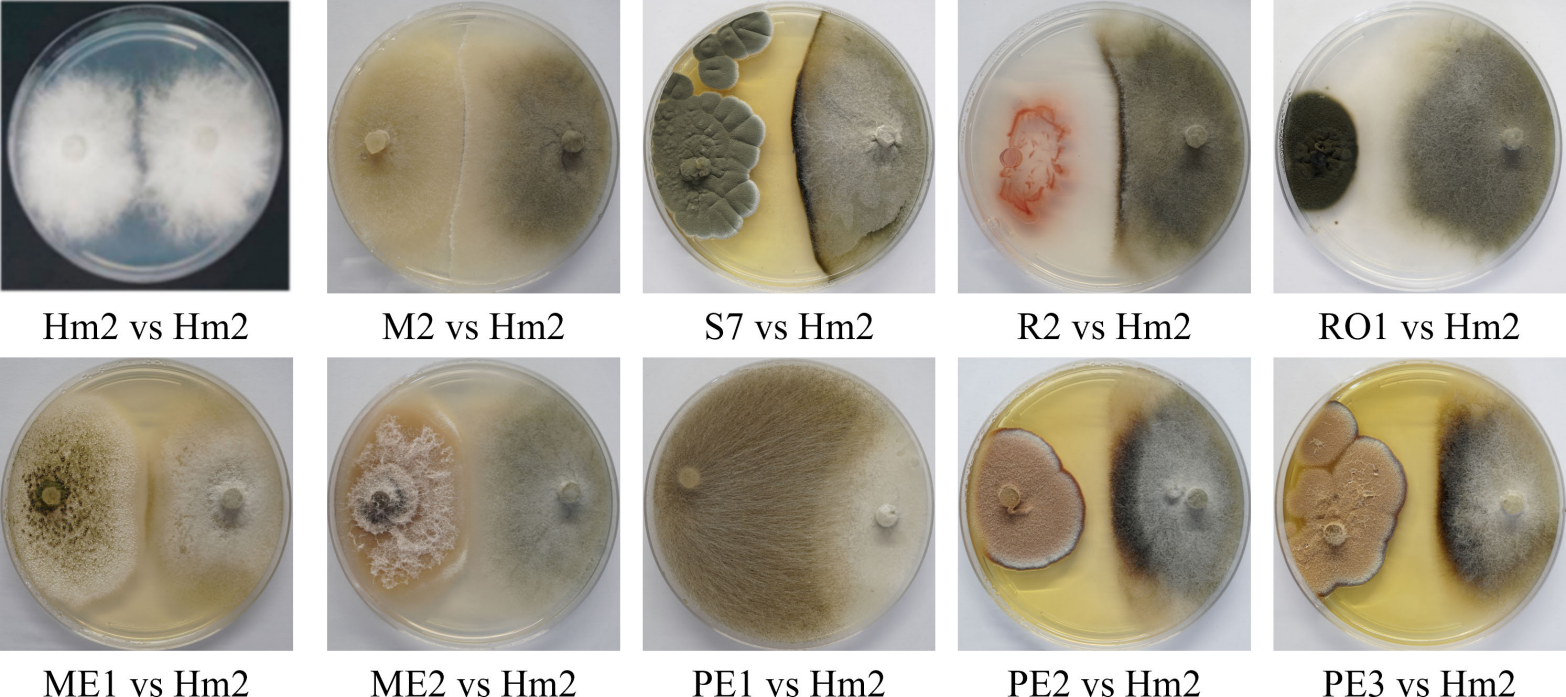
Plate confrontation assay to identify *M. maydis* antagonism. The PDA plate mycoparasitism assay after seven days of incubation was used as a direct way to identify interactions between *M. maydis* and selected endophytes studied here. Tested microorganisms are listed in **Table 3**. The control was a growth medium where the pathogen *M. maydis* seeded on both poles (Hm2 vs. Hm2). Each photo represents five repeats for each confrontation assay. Species that managed to restrict *M. maydis* growth were marked as having microparasitic potential (grow above *M. maydis* mycelium or inhibit it by hypha contact or by creating antifungal compounds). In weak antagonists, as can be seen for example in RO1 (on the left) vs. Hm2 (on the right), the *M. maydis’s* mycelium covered almost the entire dish.

**Figure 5:**
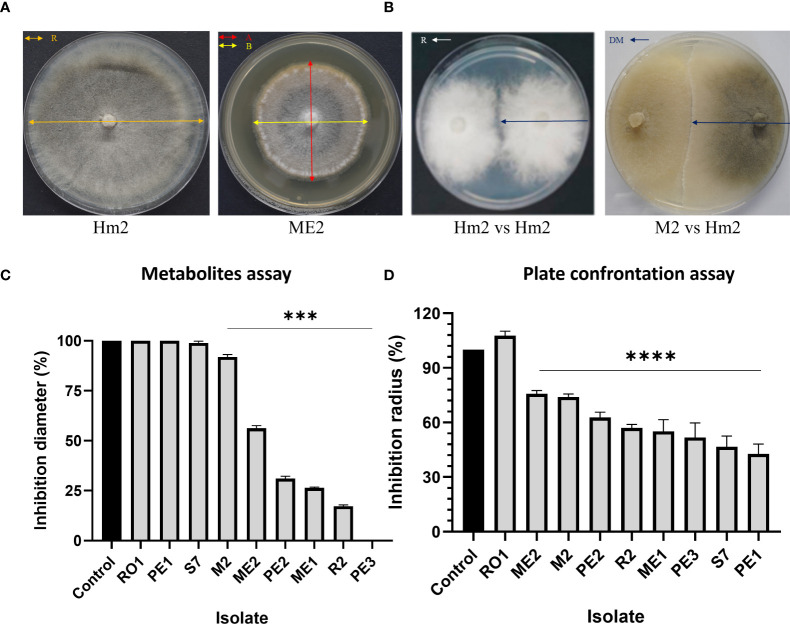
Percentage of inhibition of *M. maydis* (%I) in the metabolites (**Figure 3**) and plate confrontation (**Figure 4**) assays. The following calculation measured *M. maydis*’ inhibition **(A, B)**. For the metabolites assay **(C)**: (%I) = [((A+B)/2)x100/R], where R = radial growth of the control; A+B = radial growth of treatment. For the mycoparasitism experiment **(D)**, the calculation is (%I) = [(R-DM) x100/R], where R = radial growth of the control; DM = radial expansion of treatment (Chagas et al., 2022). The vertical upper bars represent the standard error of the mean of the 5-6 replicates. Asterisks indicate a significant difference from the control [*p* < 0.0005 (***), < 0.00005 (****)].

On solid media, after six days, the seed colonists *M. maydis* confrontation assay results (**Figure 4**) supported the above results. This examination revealed that some endophytes could restrict *M. maydis* colony growth by secreting their extrolites (for example, S7, R2, PE2, PE3) or by physical hypha-hypha contact (for instance, *Chaetomium cochliodes*, M2). One isolate, *Mucor circinelloides* (PE1), grew above the *M. maydis* colony and inhibited its growth. Unexpectedly, this species was unsuccessful in the metabolites assay (**Figure 3**). In contrast, some isolates such as RO1 were poor *M. maydis* antagonists in both assays. Quantitative analysis of both the extrolites and confront experiments is presented in **Figure 5**. The highest inhibition scores (%I) were for *Aspergillus terreus* (PE3) in the metabolites assay and *Mucor circinelloides* (PE1) in the mycoparasitism experiment. An intriguing result regards S7, which was unsuccessful in the metabolites assay but showed strong non-direct antagonism in plate confrontation against *M. maydis*. Bio-protective species that excel in both trials (*p* < 0.005) are ME1 (*A. flavus*), ME2 (*Fusarium* sp.), PE2, PE3 (*A. terreus*), and the control species R2 (*B. subtilis*) and M2 (*C. cochliodes*). The newly discovered species were further examined in the seedlings’ pathogenicity assay.

**Figure 6:**
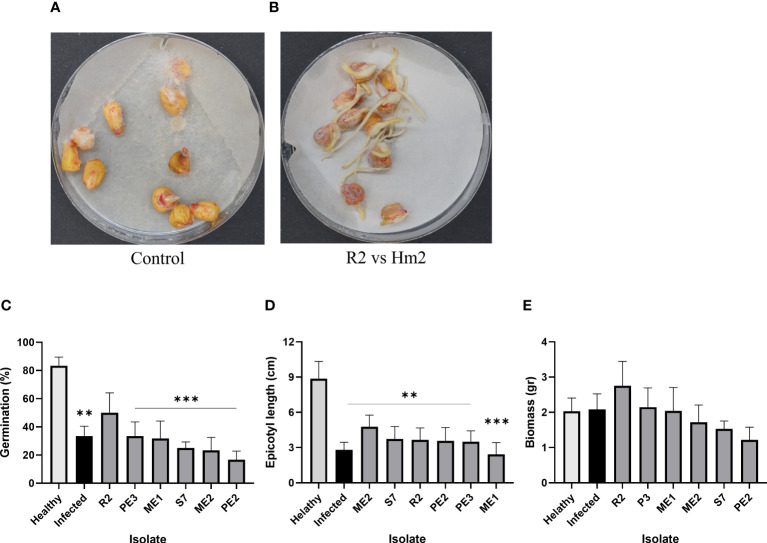
Seedlings pathogenicity assay. The inhibition influence of *M. maydis* on the seeds’ germination and the sprouts’ first development *in vitro*. The seeds were enriched by the endophytes’ secreted metabolites (15 min incubation in their growth fluid). **(A)** The control plate included *M. maydis* stressed seeds (Infected) and mock uninfected seeds (Healthy); **(B)** representative plate of *B subtilis* (R2) versus *M. maydis* (Hm2). Six endophytes were added separately to the seeds *in vitro*. Seeds germination percentage **(C)**, epicotyl length **(D)** and sprouts’ fresh biomass **(E)** were measured six days after incubation at 28 ± 1°C in the dark. Vertical upper bars represent the standard error of the mean of six replicates (Petri dishes, each containing 10 seeds). Asterisks indicate a significant difference from the mock control [*p* < 0.005 (**), < 0.0005 (***)].

The seeds’ endophytes that passed both the metabolites and the confront antagonism tests were selected for a seedling pathogenicity assessment. The germination values of the LWD susceptible sweet maize Prelude cv. seeds enriched with the protective endophytes were measured under the pathogen stress in Petri dishes (**Figure 6**). While statistical significance could only be reached compared to the non-infected control, all the endophytes studied (except ME1) enhanced the germinating seeds’ epicotyl elongation (23% or more), with *Fusarium* sp. (ME2) being the most successful (reaching 43% enhancement over the infected control). Also, *B. subtilis* (R2) evidently promoted the seeds’ germination (by 50%) and the sprouts’ biomass (by 34%) compared to the infected control. In contrast, other species (particularly *A. terreus*, PE2) caused some decrease in those measures (up to 50% and 41% reduction compared to this control).

## 4 Discussion

Late wilt disease (LWD), caused by the fungus *Magnaporthiopsis maydis*, threatens commercial maize production in high-risk areas. Searching for control options against the pathogen is one of the top priorities in Israel, Egypt and other countries. While disease-resistant maize genotypes can reduce yield loss, aggressive variants of the fungus can overcome host resistance (Ortiz-Bustos et al., 2015; Shofman et al., 2022). Among alternative ways to control LWD, one pioneering method is to isolate, identify and study the maize seeds’ microflora bioprotective potential against LWD (Degani et al., 2021). This study aimed at expanding this research direction and enriching our understanding of the maize seeds’ natural micro-inhabitants. The results may uncover the poorly understood pathogen-pathogen crosstalk that affects LWD and assist in developing an eco-friendly method based on strengthening the bio-shielding members in those communities and, thus, the seeds’ immunity to LWD.

The first indication of endophytes-*M. maydis* interactions may be drawn from the plate’s confrontation assay. A typical response between two species that inhibit each other’s growth on an artificial rich solid medium is the formation of a dark border line between them (Mallett and Hiratsuka, 1986). The appearance of hyphal granules, dark gel-like structures, and vacuoles observed in fungal interactions may indicate cell death due to mycoparasitism or nutrient deprivation due to competitive interactions. A brown-black line demarking the combating fungal strains could be linked with melanin, 1,8-dihydroxynaphthalene (DHN), a defense against environmental stresses (Krause et al., 2020). For example, it was reported that in *Armillaria mellea*, melanized hyphal cells derived from different fungal species of complex constituted the black line (Mallett and Hiratsuka, 1986). This demarcation area was flanked on each side by vesicular cells forming the pseudosclerotial plates characteristic of each respective species.

In this study, such a dark border line was formed between *M. maydis* and *P. citrinum* (S7) and *B. subtilis* (R2), implying antagonism between these two fungi and the maize pathogen. Both endophytes have been well-studied for their ability to produce fungal-inhibiting secretions and promote plant growth. The fungus *P. citrinum* is commonly found in plants such as soybean and wheat. It produces mycotoxin citrinin, digestive enzymes (cellulase, endoglucanase and xylulase), and plant growth hormones (as summarized by (Khan et al., 2008)). The *Bacillus* species produces various antifungal compounds that suppress or kill fungal pathogens, making these bacteria popular for the biocontrol of plant diseases (Wu et al., 2015). These compounds include non-ribosomal cyclic lipopeptides and volatile organic compounds (VOCs) having strong antifungal activities (Zhang et al., 2020). In line with this, the secreted metabolites assay results show that *B. subtilis* (R2) extrolites strongly inhibit *M. maydis* growth.

Indeed, an important endophytic bacterial species that has been studied extensively is *B. subtilis (* Gond et al., 2015 *).* This bacterial genus is commonly found in the seeds of various maize varieties and can be transmitted vertically from one plant generation to the next, similar to other endophytes (Rodriguez et al., 2009). This suggests that endophytes play a crucial role in the survival of their host plants. These findings support the idea that LWD-resistant maize genotypes acquire and inherit endophytes, which significantly enhance their immunity to the *M. maydis* pathogen.

Indeed, the identified partners in maize seeds’ natural microflora, such as *B. subtilis* reported here, can resist and even reduce the pathogen’s development and spread inside the host roots. Besides their antifungal properties, bacteria can live within the plants as symbiotic endophytes, such as *Bacillus amyloliquifaciens* and *B. subtilis*, which are naturally found in many maize varieties. Research indicates that these bacteria can help protect their host plants by producing antifungal lipopeptides such as subtilomycin (Deng et al., 2019). These compounds inhibit pathogens and trigger the activation of the plant’s pathogenesis-related genes, enhancing the plant’s systemic acquired resistance (Gond et al., 2015). Specifically, a mixture of *B. subtilis* and *Pseudomonas koreensis* produced siderophores and exhibited antagonistic activity against *M. maydis (* Ghazy and El-Nahrawy, 2020 *).* Additionally, this combination prevented both pre-emergence and post-emergence damping-off and promoted plant growth under greenhouse conditions. The treatment was also highly effective in field trials, reducing infections and increasing crop yield (Ghazy and El-Nahrawy, 2020).

The results of this study support global efforts and are promoting the use of beneficial maize endophytes as a bio-barrier and protective shield against the LWD fungus. Similar to the current study, seed treatments using bio-control formulations (*B. subtilis*, *Bacillus pumilus*, *Pseudomonas fluorescens*, *Epicoccum nigrum*) have been recommended for controlling maize LWD and have shown promising results in field tests (Hamza et al., 2013). These treatments were applied over two seasons and successfully reduced the impact of *M. maydis* on pre-emergence damping-off, disease incidence and crop yield. In another study (El-Mehalowy et al., 2004), rhizosphere actinomycetes (*Streptomyces graminofaciens*, *S. rochei*, *S. annulatus*, *S. gibsonii*), yeasts (*Candida glabrata*, *C. maltosa*, *C. slooffii*) and the fungus *Rhodotorula rubra* significantly inhibited the growth of *M. maydis in vitro* and in seed treatments under controlled greenhouse conditions. When these species were applied without the LWD pathogen, they significantly improved maize plant growth parameters.

Another potential bio-shielding fungus is *P. citrinum* – a seed-borne protective fungal endophyte having a worldwide distribution (Ahmad et al., 2010b; Goko et al., 2021) and a producer of a wide range of fungal extrolites. Currently, *P. citrinum* is being explored for its production of secondary metabolites and their associated benefits on plant growth promotion (e.g., secreted gibberellin (Khan et al., 2008)) and competitive antifungals (Wu et al., 2016). *P. citrinum* secondary metabolites are also being studied as potent molecules for drug development (Sahu et al., 2022). *P. citrinum*, among many extrolites, produces the lactone antifungal HMG-CoA reductase inhibitors mevastatin and citrinin (Ahmad et al., 2010a). Thus, testing these compounds’ biocontrol potential against the LWD pathogen would be very interesting and valuable.

Despite *P. citrinum* (S7) metabolites’ strong impact in the confront test (**Figure 4**), it unexpectedly failed to antagonize *M. maydis* growth in the metabolites assay (**Figure 3**). This result is probably affected by the assay protocol, which involved autoclave sterilization of a PDA medium consisting of *P. citrinum* growth medium instead of water. This procedure destroys the structure of proteins and other heat-sensitive compounds that may have antifungal potential. Yet, some antifungal compounds produced by the seeds’ endophytes are heat-stable. One such example is *Fusarium* sp. (ME2).

It is well known that *Fusarium* spp. produces toxins, the most prominent of which are fumonisins, aflatoxins, ochratoxin A, zearalenone, DON, and T-2 and HT-2 toxins, which are frequently co-contaminants in maize seed (Tarazona et al., 2020). One illustration of their importance in pathogen-pathogen interactions is a study dedicated to kernels and *in vitro* evaluation (Lanubile et al., 2021). The work indicated that *F. verticillioides-Aspergillus flavus* interactions in maize resulted in reduced fumonisins and aflatoxins biosynthetic gene expression profiles. Such interactions could exist here and should be explored more in future studies.

The most potent *M. maydis* antagonistic fungal species identified here (excelling in both the metabolites and the confrontation trials) are *Aspergillus terreus* (PE3) and *Aspergillus flavus* (ME1). Like *Penicillium* spp., *Aspergillus* spp. represents the most chemically examined fungal group with hundreds of biologically active secondary metabolites (Liu and Versicoamides, 2017). *A. terreus* is a significant saprophytic and endophytic filamentous fungus, producing a wide variety of bioactive secondary metabolites (Amr et al., 2023). It was proposed for use as biocontrol of *Rhizoctonia solani*, the causal agent of *Phaseolus vulgaris* and *Vicia faba* damping-off disease (Abdelaziz et al., 2023). This fungal species and some other endophytes identified here may also be dormant pathogens (opportunists) waiting for the proper condition to evoke their attack on the host (Samson et al., 2011). On the other hand, *A. terreus* was found to be a plant growth promoter after its inoculation in rice and maize plants *in vivo* and *in vitro* (Javed et al., 2020). Thus, as reported here, the *A. terreus* (PE3) inhibition ability against *M. maydis* makes it a good candidate for LWD bio-friendly control.

It is important to note that many endophytes use multiple methods to combat invasive pathogens. They can directly inhibit the pathogen’s growth and also trigger the plant’s systemic defense response (Gao et al., 2010). Research into the microbial communities, both pathogenic and non-pathogenic, associated with maize could enhance crop management and yield under these threats. All maize plants in natural environments contain seed-vectored endophytes, which may affect the resistance of maize cultivars to late wilt disease (LWD). Differences in resistance between susceptible and resistant LWD maize cultivars may stem from variations in their endophytic communities.

Finally, the seeding pathogenicity assay adjusted and used in the current study is a rapid research tool for evaluating the seeds’ microflora protective members’ ability to prevent pathogens’ penetration and establishment stages. This stage is essential to rule out inefficient antagonist microorganisms, focusing research efforts on more promising ones. The results of the current study are motivating us to expand our knowledge on this subject to uncover the full potential of the maize microbiome in helping the plant survive against pathogens. Understanding these interactions under natural conditions will help us grasp (and potentially influence) the long-term effects of excluding endophyte-based biocontrol methods.

## 5 Conclusion

This study focused on understanding the endophytes’ role in seeds’ acquired resistance to late wilt disease (LWD). Most microorganism species isolated from maize grains and identified here had an antagonistic effect against the LWD pathogen *Magnaporthiopsis maydis in vitro* (extrolites and confrontation assays). The bacteria *B. subtilis* (R2) was the best protective endophyte in terms of seeds’ germination (50%) and sprouts’ biomass (34%). The maize cultivar’s resistance/susceptibility to the disease may be related to the endophyte colonizing it. Indeed, the most successful species in the confrontation and seedlings pathogenicity assays, *A. terreus* (PE2, PE3), *A. flavus* (ME1) and *B. subtilis*, were isolated from the most LWD-susceptible maize cultivars. Still, the possible link between the maize cultivar LWD susceptibility and the bioprotective potential of its endophytes community members should be clarified in future studies. Also, studying the endophytes’ impact on *M. maydis* during the later growth stages is critical. The results of this study are encouraging us to deepen and widen our understanding of this subject matter to uncover the maize microbiome’s role in the plant survival struggle against pathogens. Such future research directions may include studying the endophytes’ action mechanism involved in pathogen repression, interactions between the seeds’ microflora colonizers, and host physiology and environmental factors that affect the seeds’ microbiome composition and function. Exploring these interactions under natural conditions could help us manipulate and influence the consequences of endophyte-based biocontrol.

## Data availability statement

The original contributions presented in the study are publicly available. Data generated by DNA sequencing were deposited in the NCBI repository. GenBank accession numbers for the nucleotide sequences: HM2 *Magnaporthiopsis maydis* PP794674, Ht1 *Fusarium dhileepanii* PP794675, S2 *Fusarium dhileepanii* PP794676, S7 *Penicillium citrinum* PP794677, S9 *Fusarium dhileepanii* PP794678, R2 *Bacillus subtilis* PP811660, Vi2 *Macrophomina phaseolina* PP794679, D6 *Rhizopus arrhizus* PP794680, J1 *Rhizopus arrhizus* PP794681, P1 *Trichoderma asperellum* PP794682, M2 *Chaetomium subaffine* PP794683, M3 *Chaetomium cochliodes* PP794684, M4 *Chaetomium cochliodes* PP794685, R1 *Alternaria alternata* PP794686, ME1 *Aspergillus flavus* PP794687, ME2 *Fusarium ananatum* PP794688, PE1 *Mucor circinelloides* PP794689, PE2 *Aspergillus terreus* PP794690, PE3 *Aspergillus terreus*PP794691, RO1 *Cladosporium cladosporioides* PP794692. All other data generated or analyzed during this study are included in this published article (and its **Supplementary Information Files**).

## Author contributions

OD: Writing – review & editing, Writing – original draft, Visualization, Validation, Supervision, Resources, Project administration, Methodology, Investigation, Funding acquisition, Formal analysis, Data curation, Conceptualization. AA: Data curation, Formal analysis, Writing – review & editing, Visualization, Validation, Methodology, Investigation. ED: Writing – review & editing, Visualization, Validation, Methodology, Investigation, Formal analysis, Data curation, Conceptualization. AG: Conceptualization, Data curation, Formal analysis, Investigation, Methodology, Validation, Visualization, Writing – review & editing.
