# Supplementary material for: Antagonistic interactions between maize seeds microbiome species and the late wilt disease agent, Magnaporthiopsis maydis
Source: Front Fungal Biol. 2024 Aug 7;5:1436759. doi: 10.3389/ffunb.2024.1436759 (PMC11337106; doi:10.3389/ffunb.2024.1436759)
Supplement: Supplementary file 1 [file DataSheet_1.docx]

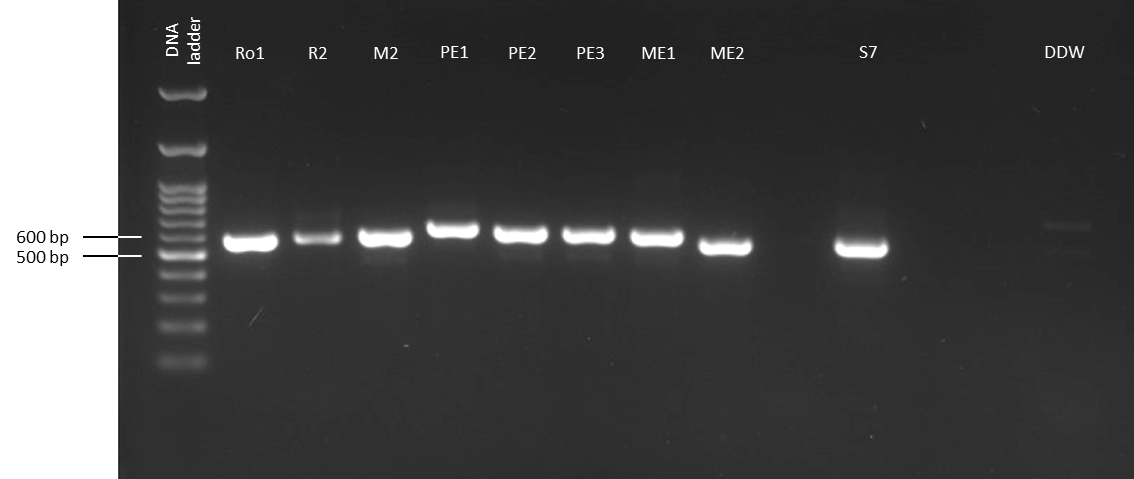


**Figure S1**. Full-length gels for the maize seeds endophytes that are shown in Figure 1. The PCR reaction for the universal internal transcribed spacer (ITS) gene product was detected by gel electrophoresis. The negative control (DDW) includes sterile twice-distilled water instead of the DNA template.

**Figure S2**. Phylogenetic analysis of the ITS4 gene of the endophytes’ isolates (presented in Table 3). The SeaView version 5.05 program (<http://doua.prabi.fr/software/seaview>, accessed on 15 May 2024) generated the phylogenetic tree using the parsimony DNA-level algorithm (dnapars)-based method with the default parameters and bootstrap with 1000 replications. The analysis contains the ITS gene from reference strains from a previous work (Degani et al., 2021) to present all maize seeds’ endophyte species identified in Israel so far and others selected from the GenBank to assist the taxonomic assignment. These can be identified by their NCBI accession number. The scale describes the genetic resemblance percentages between sequences. The number of bootstrap replications out of 100 (with the definition of 10 number thresholds to avoid a small data set effect) is presented above each node and indicates the bootstrap value. It represents the phylogenetic confidence of the tree topology. In other words, these values indicate how many times out of 100, the same branch was observed when repeating the phylogenetic reconstruction on a data set. Generally, a bootstrap value above 70 is considered a plausible score for branch validation.
